# Elevated PCSK9 Levels in Untreated Patients With Heterozygous or Homozygous Familial Hypercholesterolemia and the Response to High‐Dose Statin Therapy

**DOI:** 10.1161/JAHA.112.000028

**Published:** 2013-04-24

**Authors:** Frederick Raal, Vanessa Panz, Andrew Immelman, Gillian Pilcher

**Affiliations:** 1 Carbohydrate and Lipid Metabolism Research Unit, Department of Medicine, University of the Witwatersrand, Johannesburg, South Africa (F.R., V.P., A.I., G.P.)

**Keywords:** familial hypercholesterolemia, LDL‐cholesterol, PCSK9, statin therapy

## Abstract

**Background:**

Proprotein convertase subtilisin kexin type 9 (PCSK9) is an enzyme that impairs low‐density lipoprotein cholesterol (LDL‐C) clearance from the plasma by promoting LDL receptor degradation. Patients with familial hypercholesterolemia (FH) have reduced or absent LDL receptors and should therefore have elevated PCSK9 levels.

**Methods and Results:**

Fasting lipograms and PCSK9 levels were measured 51 homozygous FH (HoFH), 20 heterozygous FH (HeFH), and 20 normocholesterolemic control subjects. Levels were repeated following high‐dose statin therapy. LDL‐C levels were significantly higher in untreated HoFH (13.4±0.7 mmol/L) and HeFH patients (7.0±0.2 mmol/L) compared with controls (2.6±0.1 mmol/L) (*P*<0.01). Statin therapy decreased LDL‐C levels from 13.4±0.7 to 11.1±0.7 mmol/L in HoFH and from 7.0±0.2 to 3.6±0.2 mmol/L in HeFH patients (*P*<0.01). PCSK9 levels were higher in untreated HoFH (279±27 ng/mL) and HeFH (202±14 ng/mL) than in controls (132±10 ng/mL) (both *P*<0.01). High‐dose statin therapy increased PCSK9 levels from 279±27 to 338±50 ng/mL in HoFH, and significantly so in the HeFH patients from 202±14 to 278±20 ng/mL (*P*<0.01). Linear regression analysis showed a correlation between PCSK9 and LDL‐C (r=0.6769; *P*<0.0001); however, this was eliminated following statin therapy (r=0.2972; *P*=0.0625).

**Conclusions:**

PCSK9 levels are elevated in untreated FH patients, particularly in those with HoFH. High‐dose statin therapy further increases PCSK9 levels. PCSK9 inhibitors might be a beneficial therapy for FH patients, even in those with HoFH.

## Introduction

Familial hypercholesterolemia (FH) is an inherited autosomal dominant disorder characterized by extremely high levels of low‐density lipoprotein cholesterol (LDL‐C) and premature atherosclerosis. Individuals with the more severe homozygous form of FH (HoFH) develop clinically significant cardiovascular disease in early childhood and, if untreated, they rarely survive beyond the age of 30 years, whereas in those with the less severe heterozygous form (HeFH) the onset of significant cardiovascular disease is generally delayed until the fourth or fifth decade.^1^

In the majority of patients FH is due to mutations in the LDL receptor. More than 1600 mutations have been described to date and the majority are “receptor‐defective” (2% to 25% residual LDL receptor activity) rather than “receptor‐negative” (<2% residual receptor activity).^2^ Homozygous FH patients who are receptor‐negative tend to have higher LDL‐C levels, respond less well to statin therapy, and have a worse prognosis than those who are receptor‐defective.^3^ Only 5% to 10% of patients have mutations in the ligand‐binding domain of apolipoprotein B (Apo‐B), the protein component of LDL‐C that interacts with the LDL receptor.^4^ Over the past decade, however, FH has been linked to another gene that encodes the enzyme, proprotein convertase subtilisin kexin type 9 (PCSK9).^5–6^ In general, the prevalence of PCSK9 mutations is very low compared with defects in the LDL receptor and Apo‐B.^7^

PCSK9 is a serine protease synthesized mainly by the liver which binds to hepatic LDL receptors. It regulates plasma LDL‐C levels by diverting cell surface LDL receptors to lysosomes for degradation. In so doing, PCSK9 prevents the normal recycling of LDL receptors back to the cell surface. This process results in reduced LDL receptor density, decreased clearance of LDL‐C, and, consequently, accumulation of LDL‐C in the circulation.^8^ Thus PCSK9 levels tend to correlate directly with LDL‐C levels.^9^

Several mutations in the PCSK9 gene have been identified which are associated with either a hypocholesterolemic or hypercholesterolemic phenotype. “Loss‐of‐function” mutations decrease LDL receptor degradation and patients with these mutations have low LDL‐C concentrations and appear to be protected from coronary heart disease.^10^ Conversely, “gain‐of‐function” mutations accelerate LDL receptor degradation and carriers of these mutations present with elevated LDL‐C levels and heightened cardiovascular risk.^5^ In these patients, the clinical features are similar to those observed in FH patients with LDL receptor mutations.

Statins, the cholesterol‐lowering drugs that inhibit hydroxymethylglutaryl (HMG) Co‐A reductase, are widely prescribed for the treatment of FH. A meta‐analysis of data from numerous randomized trials of statins has established that reducing LDL‐C delays cardiovascular events and prolongs survival.^11^ Statins reduce LDL‐C by inhibiting the rate‐limiting step in hepatic cholesterol synthesis, resulting in increased expression of hepatic LDL receptors and increased clearance of circulating LDL‐C.^12^ However, statins do not only upregulate LDL receptors, they also simultaneously upregulate expression and secretion of PCSK9 via a process involving the sterol regulatory element‐binding protein‐2 (SREBP‐2) transcription factor.^13^ Secreted PCSK9 decreases the number of LDL receptors in hepatocytes, and in livers of parabiotic mice and knockout mice lacking PCSK9, there is an inverse relationship between PCSK9 expression and LDL receptor levels.^14–15^ If the numbers of LDL receptors are reduced as occurs in HeFH and to a much greater extent in HoFH, one would expect PCSK9 levels to be increased.

The aims of the study were to measure PCSK9 levels in untreated HoFH and HeFH patients, and to determine the effect of high‐dose statin therapy on the relationship between PCSK9 and LDL‐C levels.

## Patients and Methods

### Patients

Patients with FH were recruited from the lipid clinic at the Charlotte Maxeke Johannesburg Academic Hospital. A total of 51 HoFH patients, 20 HeFH patients, and 20 normolipidemic control subjects with no history of hypercholesterolemia or cardiovascular disease were studied. Early morning fasting lipograms and PCSK9 levels were measured in all subjects. A subset of 20 HoFH patients and 20 HeFH patients were then treated with high‐dose statin therapy, either 80 mg atorvastatin or 40 mg rosuvastatin daily, and blood samples were repeated after at least 4 weeks of statin therapy. The patients and control subjects gave informed consent and the study was approved by the Committee for Research on Human Subjects of the University of the Witwatersrand.

Criteria for the diagnosis of HoFH were genetic confirmation of 2 mutant alleles at the LDL receptor gene locus; or an untreated LDL‐C >13.0 mmol/L together with either cutaneous or tendinous xanthoma before 10 years of age, or evidence of elevated LDL‐C >4.9 mmol/L before lipid‐lowering therapy consistent with HeFH in both parents. Diagnosis of HeFH was based on genetic confirmation of a FH LDL receptor mutation; or a family history of hypercholesterolemia together with clinical signs of HeFH. LDL receptor gene mutations, which were determined as described previously.^16^

### Methods

#### Biochemical assays

Blood samples were taken in the morning after a 12‐hour overnight fast. Serum concentrations of PCSK9 were determined using an enzyme‐linked immunosorbent assay (ELISA) targeting human PSCK9 (Quantikine, R&D Systems). Lipid profiles, consisting of serum total cholesterol (TC), triglycerides (TG), and high‐density lipoprotein cholesterol (HDL‐C), were measured with standard enzymatic methods and reagents (Roche Diagnostics GmbH). LDL‐C levels were calculated using the Friedewald formula.^17^ Intra‐assay coefficients of variance were <5.0% for all assays.

#### Measurement of Carotid Intima Media Thickness (CIMT)

The carotid arteries of patients and control subjects were evaluated in the supine position with high resolution B‐mode ultrasonography using a previously validated technique.^18^ In brief, both common carotid arteries were scanned longitudinally to visualize the intima‐media complex of the far wall of the artery. The distance between the echo arising from the lumen‐intima interface and the media‐adventitia interface was taken as a measure of the intima‐media complex. CIMT was defined as the average of 5 measurements randomly selected between 10 and 30 mm proximal to the carotid bifurcation. All measurements were performed by the same technician who was blinded to the patients' drug therapy, to any previous ultrasound findings, and to whether subjects were from the HoFH, HeFH, or control group. Using this technique, the intraobserver variation was 6.7%.

### Statistical Analysis

Data analyses were performed with GB‐STAT (Dynamic Microsystems, Inc) and significance was defined as *P*<0.05. The comparison groups were HoFH patients (untreated and treated) (n=20), HeFH patients (untreated and treated) (n=20), and control subjects (n=20). One‐way analysis of variance (ANOVA) was used to compare differences across the untreated patient groups and control subjects. Pair‐wise comparisons of untreated HoFH patients and control subjects, and untreated HeFH patients and control subjects were determined with the unpaired *t*‐test for age, BMI, CIMT, TC, TG, HDL‐C, LDL‐C, and PCSK9. Pair‐wise comparisons of untreated and treated HoFH patients, and untreated and treated HeFH patients were determined using the paired *t*‐test for TC, TG, HDL‐C, LDL‐C, and PCSK9. Bonferroni paired and multiple comparison *t*‐tests were applied where appropriate. Untreated and treated values of LDL‐C and PCSK9 were used to calculate percentage changes. Multiple regression analysis of the combined groups (n=60) of untreated HoFH patients, untreated HeFH patients, and control subjects was performed using PCSK9 as the dependent variable and age, BMI, CIMT, TC, TG, HDL‐C, and LDL‐C as the independent variables. Simple linear regression was also done in this group (n=60) using the same variables before high‐dose statin therapy. The analysis was repeated after statin therapy in only the treated HoFH patients and treated HeFH patients (n=40) using PCSK9 as the dependent variable and TC, TG, HDL‐C, and LDL‐C as the independent variables. Correlation coefficients (r) were calculated to quantify the associations between the variables. Results are expressed as mean±SEM.

## Results

### Genetic Mutations

Of the 51 HoFH patients who underwent genetic testing, all but 3 had a molecular diagnosis of HoFH. Except for one patient who had autosomal recessive hypercholesterolemia, all of the patients had both LDL receptor mutations identified. The most frequent alleles were D206E (FH Afrikaner‐1), V408M (FH Afrikaner‐2), and D154N (FH Afrikaner‐3). The majority of the HoFH patients were LDL‐ receptor‐defective and only 3 were LDL‐receptor‐negative.

### Biochemical Assays

Mean concentrations of lipids and PCSK9 in a total of 51 untreated HoFH patients were as follows: TC 16.5±0.5 mmol/L, TG 1.4±0.2 mmol/L, HDL‐C 0.9±0.04 mmol/L, LDL‐C 14.9±0.5 mmol/L, and PCSK9 283±18 ng/mL.

Table 1 summarizes the clinical characteristics and mean fasting biochemical variables of the control subjects as well as those of the subset of 20 HoFH patients and 20 HeFH patients before and after statin therapy. HoFH patients were younger than the control subjects (*P*<0.01) and BMI was similar in all groups. CIMT values were higher in the HoFH and HeFH patients compared to control subjects (*P*<0.01). As expected, TC concentrations were significantly higher in the untreated HoFH and untreated HeFH patients than in control subjects (both *P*<0.01). High‐dose statin therapy decreased TC concentrations in HoFH patients and HeFH patients (both *P*<0.01). TG levels were similar in untreated and treated HoFH and HeFH patients and control subjects. HDL‐C levels were significantly lower in untreated HoFH patients compared with control subjects (*P*<0.01). LDL‐C concentrations were significantly higher in untreated HoFH patients (13.4±0.7 mmol/L) and untreated HeFH patients (7.0±0.2 mmol/L) than control subjects (2.6±0.1 mmol/L) (both *P*<0.01). The reduction in LDL‐C concentration on statin therapy showed an absolute mean decrease of 2.3 mmol/L in HoFH patients (from 13.4±0.7 to 11.1±0.7 mmol/L; *P*<0.01). In treated HeFH patients the reduction was 3.4 mmol/L (from 7.0±0.2 to 3.6±0.2 mmol/L; *P*<0.01). PCSK9 levels were also significantly higher in untreated HoFH patients (279±27 ng/mL) and untreated HeFH patients (202±14 ng/mL) compared with control subjects (132±10 ng/mL) (both *P*<0.01). However, PCSK9 levels increased in both groups of patients on high‐dose statin therapy (from 279±27 to 338±50 ng/mL in HoFH patients), and significantly so in the HeFH patients (from 202±14 to 278±20 ng/mL; *P*<0.01).The percentage increase was only 21% in HoFH patients compared with 37% in HeFH patients.

**Table 1.** Clinical Characteristics and Fasting Biochemical Variables of Patients With Homozygous or Heterozygous Familial Hypercholesterolemia Before and After Statin Therapy, and Control Subjects

	Homozygous FH Patients (n=20)		Heterozygous FH Patients (n=20)		Control Subjects (n=20)	Untreated Patients vs Controls (*P* Value)*	Untreated vs Treated Patients (*P* Value)*
	Untreated	Treated	Untreated	Treated			
Gender (M/F)	13/7	—	10/10	—	7/13	—	—
Age, y	25.7±1.6	—	41.4±3.0	—	42.4±2.3	<0.01	—
BMI, kg/m^2^	24.0±1.0	—	25.0±1.0	—	23.1±0.6	NS	—
CIMT, mm	1.4±0.11	—	0.7±0.04	—	0.5±0.02	<0.01	—
Total cholesterol, mmol/L	15.0±0.7	12.9±0.8	9.2±0.2	5.7±0.2	4.8±0.1	<0.01	<0.01
Triglycerides, mmol/L	1.5±0.3	1.5±0.2	1.7±0.2	1.4±0.2	1.2±0.1	NS	NS
HDL‐C, mmol/L	0.9±0.1	1.1±0.1	1.4±0.1	1.5±0.1	1.6±0.1	<0.01	NS
LDL‐C, mmol/L	13.4±0.7	11.1±0.7	7.0±0.2	3.6±0.2	2.6±0.1	<0.01	<0.01
PCSK9, ng/mL	279±27	338±50	202±14	278±20	132±10	<0.01	<0.01

Results are expressed as mean±SEM. Conversion factors: total cholesterol, HDL‐C, LDL‐C (mmol/L)÷0.0259=mg/dL; triglycerides (mmol/L)÷0.0113=mg/dL. FH indicates familial hypercholesterolemia; M, male; F, female; BMI, body mass index; CIMT, carotid intima media thickness; HDL‐C, high‐density lipoprotein cholesterol; LDL‐C, low‐density lipoprotein cholesterol; PCSK9, proprotein convertase subtilisin kexin type 9; NS, not significantly different; HoFH, homozygous familial hypercholesterolemia; HeFH, heterozygous familial hypercholesterolemia.

* *P*<0.01; untreated HoFH or untreated HeFH patients vs control subjects except for age and HDL‐C in untreated HeFH patients vs control subjects (*P*>0.05, NS).

* *P*<0.01; untreated vs treated HoFH patients or untreated vs treated HeFH patients except for PCSK9 in untreated vs treated HoFH patients (*P*>0.05, NS).

**Figure 1.**
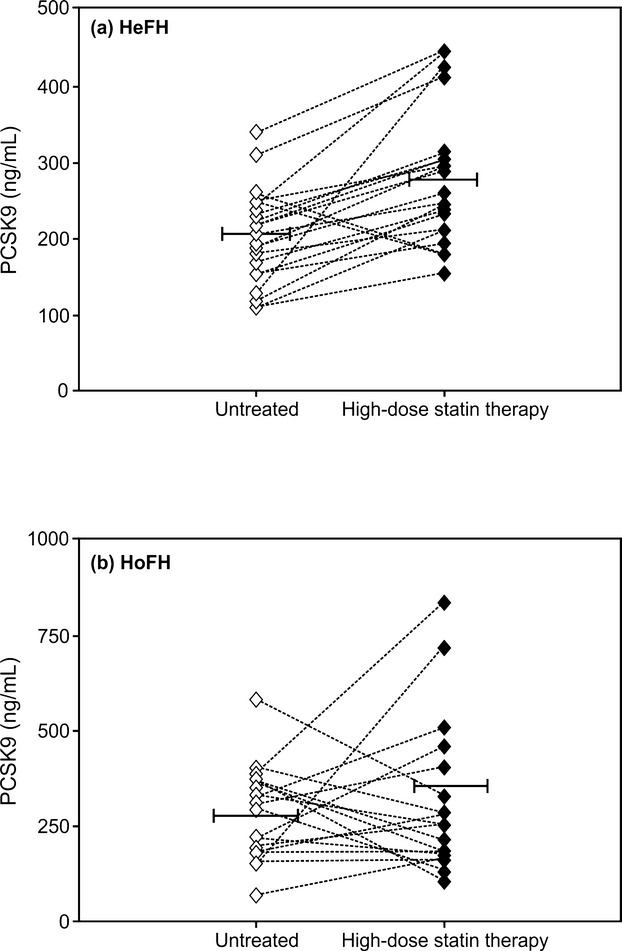
Ladder plots showing paired levels of proprotein convertase subtilisin kexin type 9 (PCSK9) before and after high‐dose statin therapy in patients with (a) heterozygous familial hypercholesterolemia (HeFH; n=20), mean±SEM (ng/mL)=202±14 vs 278±20 (*P*<0.01; paired *t*‐test) and (b) homozygous familial hypercholesterolemia (HoFH; n=20), mean±SEM (ng/mL)=279±27 vs 338±50 (*P*>0.05; paired *t*‐test).

Figure 1 shows paired levels of PCSK9 in HeFH patients and HoFH patients before and after high‐dose statin therapy. PCSK9 levels increased with statin therapy in 18 of the 20 HeFH patients (*P*<0.01) and in 10 of the 20 HoFH patients (*P>*0.05).

### Regression Analysis

**Figure 2.**
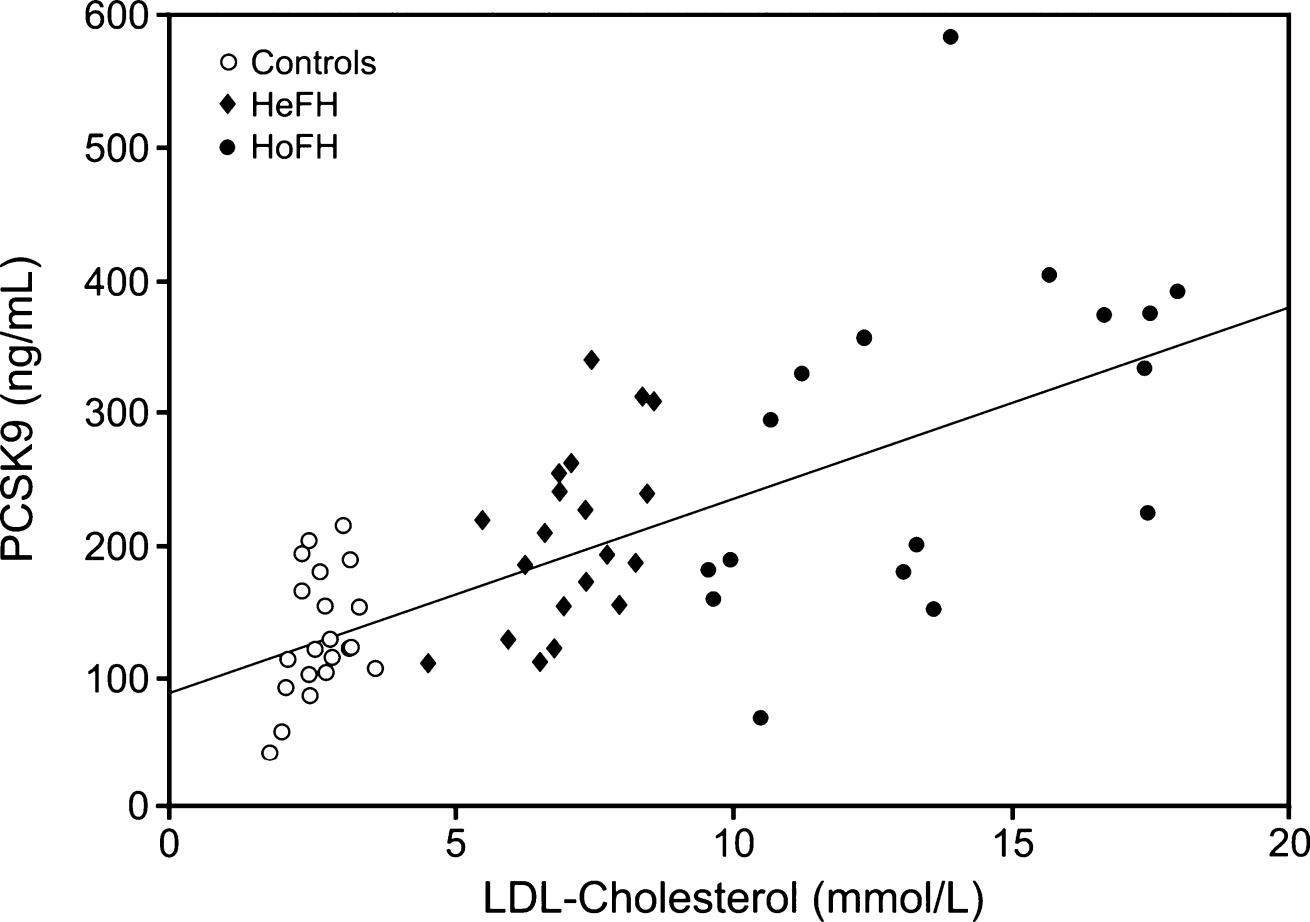
Linear regression plot showing the correlation between PCSK9 and LDL‐cholesterol in the combined groups of untreated patients with heterozygous familial hypercholesterolemia (HeFH; n=20), homozygous familial hypercholesterolemia (HoFH; n=20) and control subjects (n=20) (r=0.6769; *P*<0.0001). PCSK9 indicates proprotein convertase subtilisin kexin type 9; LDL, low‐density lipoprotein.

Multiple regression analysis of the combined groups of untreated patients and control subjects, using PCSK9 as the dependent variable and age, BMI, CIMT, TC, TG, HDL‐C, and LDL‐C as independent variables, found the greatest level of significance with LDL‐C (r=0.7282; *P*<0.0001). Simple linear regression analysis showed that the highest correlations were between PCSK9 and LDL‐C (r=0.6769; *P*<0.0001) (Figure 2), PCSK9 and TC (r=0.6745; *P*<0.0001), and PCSK9 and CIMT (r=0.3916; *P*<0.0022). However, high‐dose statin therapy eliminated the significant positive correlations between PCSK9 and TC (r=0.2922; *P*= 0.0673) and LDL‐C (r=0.2972; *P*= 0.0625) (Table 2). There was no correlation between the percentage change (reduction) in LDL‐C and the percentage change (increase) in PCSK9 levels in the HoFH group (r =−0.0671; *P*=0.7850), the HeFH group (r=0.1152; *P* =0.6387), or the combined patients as a group (r =−0.0838; *P*=0.6171).

**Table 2.** Correlations Between PCSK9 and Individual Variables in Combined Groups of Patients With Homozygous Familial Hypercholesterolemia, Heterozygous Familial Hypercholesterolemia and Control Subjects Before High‐Dose Statin Therapy and Without Control Subjects After High‐Dose Statin Therapy

Variable	Untreated (n=60)		After High‐Dose Statin Therapy (n=40)	
	r	*P* Value	r	*P* Value
Age	−0.2902	0.0258	—	—
BMI	0.1119	0.3987	—	—
CIMT	0.3916	<0.0022	—	—
TC	**0.6745**	**<0.0001**	**0.2922**	**0.0673**
TG	−0.1096	0.4087	−0.2193	0.1739
HDL‐C	−0.1966	0.1355	0.0957	0.5571
LDL‐C	**0.6769**	**<0.0001**	**0.2972**	**0.0625**

Numbers shown in bold indicate that high‐dose statin therapy eliminated the significant correlations between PCSK9 and TC, and PCSK9 and LDL‐C. PCSK9 indicates proprotein convertase subtilisin kexin type 9; BMI, body mass index; CIMT, carotid intima media thickness; TC, total cholesterol; TG, triglycerides; HDL‐C, high‐density lipoprotein cholesterol; LDL‐C, low‐density lipoprotein cholesterol.

## Discussion

Findings of this study have shown that PCSK9 levels are elevated in untreated FH patients, particularly in subjects with HoFH. PCSK9 correlated positively with TC and LDL‐C; however, after statin therapy, these correlations were eliminated. High‐dose statin therapy decreased LDL‐C concentrations and increased PCSK9 levels in both groups of patients, but the percentage increase in PCSK9 was much lower in HoFH patients than in HeFH patients. Following statin therapy, the rise in PCSK9 levels was variable in HoFH patients, whereas this rise was consistent in the majority of HeFH patients. CIMT, a surrogate marker of the extent of atherosclerosis, was markedly thickened in the HoFH patients compared with HeFH patients and control subjects.

The positive correlation between PCSK9 and TC has been well described^9,19–20^ and it is the LDL‐C component of total cholesterol that influences this correlation. The elevated PCSK9 levels seen particularly in our untreated HoFH patients and to a lesser extent in untreated HeFH patients, coincided with their high LDL‐C concentrations, producing a significant positive correlation between these 2 parameters. While high‐dose statin therapy lowered LDL‐C concentrations in both groups of patients as expected, statin therapy simultaneously elevated PCSK9 levels. Consequently, the correlation between PCSK9 and LDL‐C was disrupted.

Our results are in keeping with several researchers who have reported statin‐induced increases in PCSK9 together with a loss of its correlation with LDL‐C.^21–24^ In these studies, however, the rate of increase in PCSK9 levels varied depending on the statin dose and duration of therapy. Careskey et al^21^ found that 40 mg atorvastatin/day increased PCSK9 by 34% in dyslipidemic patients and after 12 weeks of therapy the correlation between PCSK9 and LDL‐C was abolished. In a study of patients with diabetes, Cariou et al^22^ reported that PCSK9 rose by 32% on statin therapy, which destroyed the correlation between PCSK9 and LDL‐C. Following this report, Dubuc et al^23^ observed significant increases in PCSK9 levels over 12 weeks with increasing statin doses (atorvastatin from 5 to 8 mg/day and rosuvastatin from 5 to 40 mg/day), representing an overall gain of 45%. During a time‐course study performed with 80 mg atorvastatin in normolipidemic individuals, Welder et al^24^ demonstrated a similar rise of 47% in PCSK9 levels within 4 weeks. By contrast, statin therapy at maximum daily doses of 80 mg atorvastatin or 40 mg rosuvastatin for at least 4 weeks resulted in an increase of only 21% in our HoFH patients as opposed to 37% in the HeFH patients.

In view of these findings, it was not surprising that high‐dose statin therapy caused the correlation between PCSK9 and LDL‐C to be absent in our FH patients. This effect may be due to intracellular cholesterol depletion caused by statin‐induced activation of SREBP‐2, which, in turn, upregulates expression of both the LDL receptor and PCSK9 genes, leading to elevated circulating levels of PCSK9.^25^ Interestingly, PCSK9 levels increased variably in response to statin therapy in only 50% of HoFH patients, whereas levels rose steadily in the majority of HeFH patients. A significant relationship was not observed between the LDL‐C reduction and the increase in PCSK9 levels. This could be explained by the wide individual variation among the HoFH patients. A possible interpretation of this variability in PCSK9 levels among HoFH patients is that the magnitude of statin‐induced PCSK9 increase is indicative of differences in SREBP‐2 activity and variable upregulation of PCSK9.^25^

The variability may also be related to varying degrees of LDL receptor expression. HoFH patients, for example, who are LDL‐receptor‐negative have <2% of normal LDL receptor activity, while LDL‐receptor‐defective HoFH patients have approximately 2% to 25% residual LDL receptor activity.^7^ The 20 HoFH patients in whom we measured PCSK9 levels before and after high‐dose statin therapy were all receptor‐defective. Interestingly, the response to therapy was highly variable with some patients demonstrating a decrease in PCSK9 levels and others an increase.

Elevation in PCSK9 levels further diminishes the number of LDL receptors, thereby blunting the efficacy of statins. This may explain why most LDL‐C reduction is achieved with the starting dose of a given statin and why doubling the statin dose only further reduces LDL‐C concentrations modestly.^26^ Statin‐induced increases in PCSK9 levels may, therefore, account for the less than expected incremental reduction of LDL‐C concentrations in response to escalating doses of statins.

Recently, several laboratories have developed and used various ELISAs to measure PCSK9 levels.^8,19–23^ Concentrations of PCSK9 varied widely among these assays (between 35 ng/mL and 13.3 μg/mL) depending on the population being studied; differences in antibody specificities to bind PCSK9 and the standards used in the assays. PCSK9 levels varied by ≈100‐fold in a large cohort of subjects in the Dallas Heart Study.^27^ This makes it difficult to compare our results with other studies. Nonetheless, the ELISA used in our study contains recombinant human PSCK9 and is able to accurately recognize free and LDL receptor‐bound PCSK9. In addition, the same assay was used for all 3 comparative groups, thus the relative differences in PCSK9 levels between the groups are probably correct.

At present, conventional lipid‐lowering drugs such as statins and ezetimibe deliver insufficient reductions in LDL‐C concentrations, particularly in HoFH subjects, even when these agents are administered at maximum doses. Several new agents are under investigation for their potentially beneficial treatment of HoFH. Mipomersen is an antisense inhibitor of Apo B‐100. When given to HoFH patients on maximally tolerated doses of lipid‐lowering therapy, LDL‐C decreased by an additional 25%.^3^ Lomitapide, an oral microsomal triglyceride transfer protein inhibitor, reduced LDL‐C by 50% in HoFH patients.^28^ However, the effect of these agents on PCSK9 levels is unknown.

The search for a novel lipid‐lowering therapy has recently focused on developing pharmacological approaches that block the capacity of PCSK9 to degrade LDL receptors. PCSK9 activity appears to correlate with PCSK9 levels. Monoclonal antibodies directed against PCSK9 reduce LDL‐C levels but as their inhibitory effect wanes, PCSK9 levels rise and LDL‐C concentrations tend to increase.^29^ Several monoclonal antibodies are being currently assessed in human clinical trials. A recent randomized, controlled phase 1 trial of a monoclonal antibody to PCSK9 (SAR236553/REGN727) reported decreases in LDL‐C of 41% to 58% in patients with FH.^30^ Similarly, McKenney et al observed LDL‐C reductions of 40% to 72% in patients with primary hypercholesterolemia who were receiving ongoing stable atorvastatin therapy.^31^ In a recent phase 2 study, the administration of SAR236553/REGN727 in high‐risk HeFH patients treated with high‐to‐maximum doses of statins reduced LDL‐C concentrations further by up to 68%.^32^ Results of the recent Reduction of LDL‐C with PCSK9 Inhibition in Heterozygous Familial Hypercholesterolemia Disorder (RUTHERFORD trial) of AMG 145, a fully human monoclonal antibody against PCSK9, demonstrated significant LDL‐C reductions of between 43% and 55% in HeFH patients who were already taking statin therapy.^29^

These studies indicate that PCSK9 inhibition could be an alternative monotherapy for hypercholesterolemic patients who cannot tolerate statins. It might also be an effective therapy for patients who have not reached desirable LDL‐C targets. The results of our study showed that untreated FH patients, particularly those with HoFH, had elevated PCSK9 levels. While LDL‐C concentrations decreased in response to high‐dose statin therapy as expected, PCSK9 levels in HoFH and HeFH patients increased significantly. Our findings support the view that in patients with FH, even in those with HoFH whose LDL receptors are defective, PCSK9 inhibitors used in combination with statins could reduce LDL‐C levels further and may be of added benefit in the treatment of these high‐risk patients.

## Disclosure

None.
